# Relationship between Color and Translucency of Multishaded Dental Composite Resins

**DOI:** 10.1155/2012/708032

**Published:** 2012-01-26

**Authors:** Homan Naeimi Akbar, Keyvan Moharamzadeh, Duncan J. Wood, Richard Van Noort

**Affiliations:** School of Clinical Dentistry, University of Sheffield, Claremont Crescent, Sheffield S10 2TA, UK

## Abstract

The aim of the present study was to compare the translucency of different shades of two highly aesthetic multilayered restorative composite resins. In total nine shades from Esthet.X and ten shades from Filtek Supreme composite resins were chosen. Discs of each shade were prepared (*N* = 3) and light-cured. Total and diffuse transmittance values for each sample were measured. Statistical analysis showed that the opaque dentine shades of both composites were the least translucent and the enamel shades had the highest translucency. There was a significant decrease in translucency from A2 to C2 of regular body shades and also from A4 to C4 of opaque dentine shades of Esthet.X composite resin. Grey enamel shade had a significantly higher diffuse translucency compared to clear and yellow enamel shades. There was a significant decrease in translucency from A2B to D2B and also in diffuse translucency from A4D to C6D shades of Filtek Supreme composite resin. It can be concluded that the color of the composite resins tested in this study had a significant effect on their translucency. Information on the translucency of different shades of composite resins can be very useful for the clinicians in achieving optimal esthetic restorative outcome.

## 1. Introduction

Understanding and correct analysis of the optical properties of the natural dentition such as color and translucency and the differences between the natural teeth and restorative materials are very important in accurate and consistent shade selection and the proper use of restorative materials in order to achieve clinical success [1–3].

There are a number of parameters affecting the translucency of composite restorations such as thickness [4], filler particles and opacifiers [5], and resin matrix composition [6]. Several investigators have evaluated the translucency of dental composite resins [7–13] and the influence of different other factors on the translucency such as flowability [11], light curing [14, 15], resin polymerization, and aging [16–20]. 

The relationship between color and translucency of dental composite resins has been the focus of research in the recent decade [21, 22]. It has been demonstrated that more chromatic shades of commercial resin composites are less translucent [23].Yu and Lee measured and classified the translucency of varied brands and shades of resin composites. The results of their study showed that the translucency was significantly influenced by the shade designation of resin composites. Recently, it has been reported that the color of several esthetic composite resins significantly affected their translucency to such an extent that the boundaries between the shade categories were not distinct, and there were some overlaps between the enamel and body shades [24].

The aim of this study was to compare the translucencies of different shades of two commercially available highly aesthetic multilayered restorative composite resins to establish whether there is a logical pattern to the translucency of multilayered composite resins which can be consistent with their color.

## 2. Materials and Methods

### 2.1. Materials

Two commercially available multilayered dental composite resin systems were tested in this study: Filtek Supreme (3M ESPE, USA) and Esthet.X (Dentsply, Germany).

3M ESPE Filtek Supreme Universal Restorative material is a visible-light activated restorative nanocomposite designed for use in anterior and posterior restorations. The resin system is based on BIS-GMA, BIS-EMA, and UDMA with small amount of TEGDMA. Translucent shades contain a combination of a nonagglomerated/nonaggregated, 75 nm silica nanofiller, and a loosely bound agglomerate silica nanocluster consisting of agglomerates of primary silica nanoparticles of 75 nm size fillers. The cluster size range is 0.6 to 1.4 microns. The filler loading is 72.5% by weight. The translucent shades are not radiopaque. All of the remaining shades contain nonagglomerated/nonaggregated, 20 nm nanosilica filler, and loosely bound agglomerated zirconia/silica nanocluster, consisting of agglomerates of primary zirconia/silica particles with size of 5–20 nm fillers. The cluster particle size range is 0.6 to 1.4 microns. The filler loading is 78.5% by weight. These shades are radiopaque [25].

Esthet.X Micro Matrix Restorative is an esthetic, visible-light-cured, radiopaque microhybrid composite restorative material designed specifically for use in all cavity classes in both anterior and posterior restorations. The resin matrix of Esthet.X is a urethane modified BIS-GMA resin matrix system. It consists mainly of BIS-GMA adduct, ethoxylated bisphenol-A-dimethacrylate, and TEGDMA. The filler component of Esthet.X is a blend of a proprietary inorganic bariumalumino fluoroborosilicate (BAFG) glass with nanosized silicon dioxide particles. The BAFG glass has an average filler particle size of 0.6–0.8 microns with narrow particles size distributions of 0.02–2.5 microns. The silicon dioxide nanofiller is in the range of 10 to 20 nm. The total percentage by volume of inorganic filler is ca. 60 vol.%; the percentage by weight is ca. 77 wt.% [26].

In total nine shades from Esthet.X and ten shades from Filtek Supreme were chosen.

Esthet.X shades included A4, B2, C4 opaque dentine, A2, B2, C2 regular body, clear enamel (CE), yellow enamel (YE), and gray enamel (GE) shades.

Filtek Supreme shades included A4D, A6D, C4D, C6D (dentine), A2B, C2B, D2B (body), A2E, B2E, and D2E (enamel) shades.

### 2.2. Specimen Preparation

Three disc samples of each shade were prepared (*N* = 3) by the following method. A mould was made by cutting 15.5 mm diameter holes in a 1.1 mm thick sheet of polycarbonate.This was done using a standing drilling machine (Weddings Industries UK LTD). The mould was then placed on a glass plate and the dental composite was placed inside it. The composite was uniformly packed by a condenser. A second glass plate was placed on the top of the mould and firmly pressed on the mould for twenty seconds. The composite was then cured using an Elipar TriLight light curing system. The light intensity was measured (750 mW/cm²) each time before curing to make sure the intensity of the light remained the same. The samples were cured from five different angles, each for twenty seconds.

 The thickness of each of the samples was measured by a micrometer (WPI, USA) at five different sites (four corners and at the centre). Only samples with a thickness of 1.1 ± 0.05 mm thickness were accepted.

Specimens were polished on wet 400 and 600 grit silicon carbide grinding paper (Buehler-Met II, Buehler, UK). This was followed by polishing with 6–1 *μ*m finish with diamond compounds (Buehler-met AS, USA) on a grinder-polisher (Buehler, Coventry, UK) at 200 rpm for 5 min using light hand pressure. After the finishing procedure the samples were washed with water and their thickness was remeasured to achieve a uniform thickness of 1.0 ± 0.05 mm. Each of the samples was then inspected on a light box to ensure that no porosity was present. Finally a total of fifty-seven samples were prepared to measure the translucency of each. Three samples of each shade were stored in a polybag in a dry environment.

### 2.3. Measurement of Optical Properties

Transmittance values were recorded for each sample using a UV/VIS spectrophotometer (Perkin Elmer Lambda 2) with an integrating sphere.

Standard illuminant D65 corresponding to average daylight was used.

For total transmittance measurement, a sample was placed in the transmission port (entry port) of the spectrophotometer and a white reference material was placed in the reflectance port. Total transmittance then was recorded at every wavelength from 380 nm to 700 nm.

**Figure 1:**
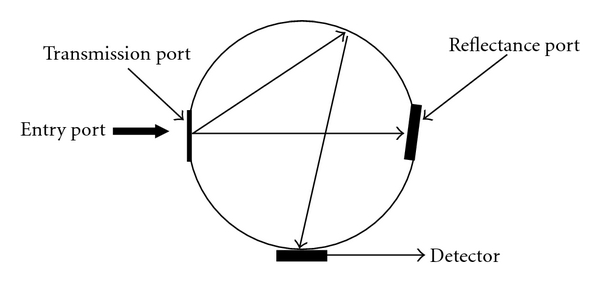
Schematic picture of the mechanism of light transmittance detection by spectrophotometer. Direct transmission can be seen traveling straight across the sphere to the reflectance port. Diffuse transmittance can be seen reflecting around the sphere.

Diffuse transmittance was measured by using a light trap in the reflectance port. A light trap can be either a black background or an open port as a light trap. The light trap absorbs the directly transmitted light; therefore only the scattered light is measured. In this study an open port was used as a light trap (Figure 1). Each sample was measured for diffuse transmittance between 380 nm to 700 nm. Average total and diffuse transmittance of each shade of the composite resins were calculated by dividing the sum of the readings from 380 nm to 700 nm by 321.

### 2.4. Statistical Analysis

Statistical analysis of the data was carried out by one-way ANOVA followed by Tukey's test using Minitab statistical analysis software.

## 3. Results

**Figure 2:**
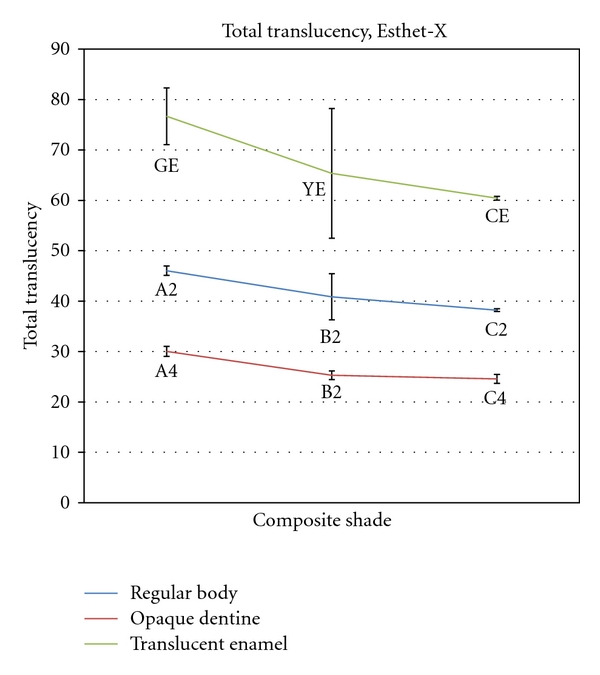
Mean total translucency of different shades of Esthet-X composite resin.

**Figure 3:**
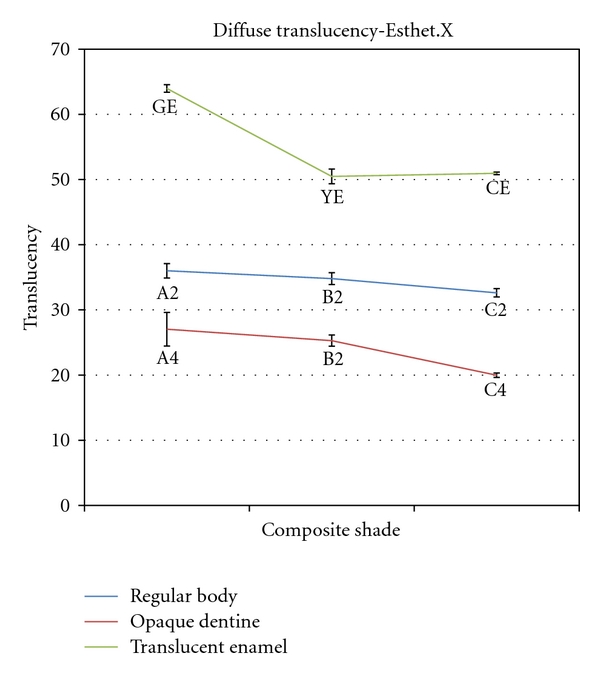
Mean diffuse translucency of different shades of Esthet-X composite resin.

Mean total and diffused transmittance values for different shades of Esthet-X dental composite resins are presented in Figures 2 and 3. Statistical analysis by one-way ANOVA followed by Tukey's test showed that the opaque dentine shades had the lowest total and diffuse transmittance values and the translucent enamel shades had the highest transmittance values in Esthet-X composite resin. Statistical analysis within the groups revealed that there was a significant decrease in both total and diffused transmittance from A2 to C2 of regular body shades and also from A4 to C4 of opaque dentine shades of Esthet-X composite resin. Within the translucent enamel group, grey enamel shade had a significantly higher diffuse translucency compared to clear and yellow enamel shades.

**Figure 4:**
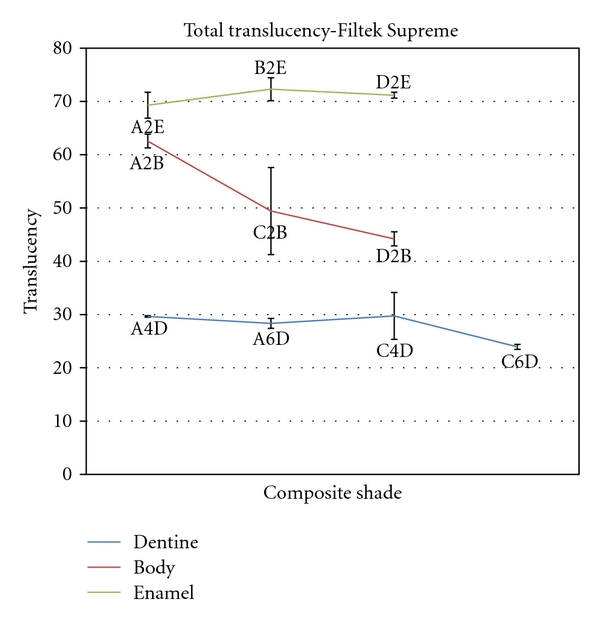
Mean total translucency for different shades of Filtek Supreme composite resin.

**Figure 5:**
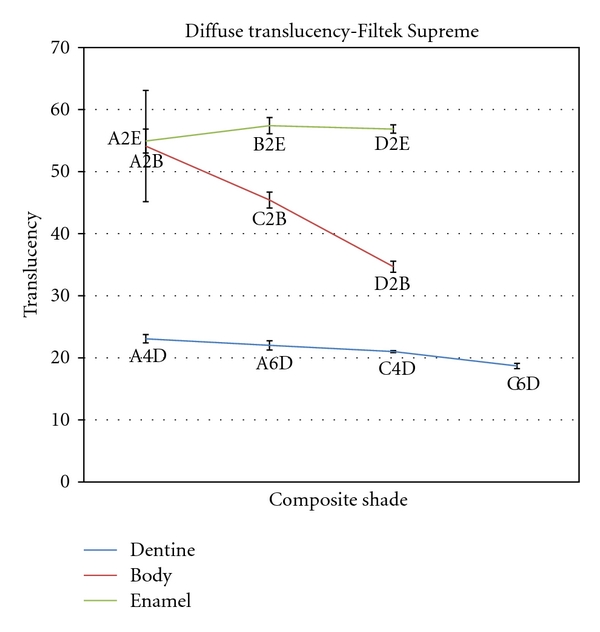
Mean diffuse translucency for different shades of Filtek Supreme composite resin.

Mean total and diffuse transmittance values for different shades of Filtek Supreme dental composite resins are shown in Figures 4 and 5. Statistical analysis by one-way ANOVA followed by Tukey's test showed that the dentine shades had the lowest total and diffuse transmittance values and the enamel shades had the highest transmittance values in Filtek Supreme composite resin. Statistical analysis within the groups revealed that there was a significant decrease in both total and diffuse transmittance values from A2B to D2B and also in diffuse transmittance from A4D to C6D shades of Filtek Supreme composite resin. However, there was no significant difference in total transmittance within the dentine shades and neither in total and diffused transmittance within the enamel shades of this composite resin.

## 4. Discussion

In the present study the translucency of different shades of two very commonly used universal composite resins was evaluated. These two systems were chosen because of their wide acceptance and popularity among clinicians.

In selection of the shades for the study care was taken to choose the shades in a way that they present a comparable sample within any given group of each resin system and also allow us to compare their optical properties of different shades within a certain group.

Opaque dentine shades had the lowest total and diffused translucency values, and the translucent enamel shades had the highest translucency values in Esthet-X composite resin. A similar pattern was observed with Filtek Supreme resin. Dentine shades had the lowest total and diffused translucency values, and the enamel shades had the highest translucency values. These findings are consistent with the findings of Kamishima et al. [27] who reported that the opaque shades of the composite resins were less translucent than other shades. Similarly, Ikeda et al. [21] demonstrated that opaque shade was less translucent than body shades of several restorative composite materials. Translucency of these materials is influenced by the difference in the refractive indices between resin matrix and filler. As mentioned above, different shade groups of the composite resins used in this study have different compositions in terms of filler contents and nanofiller particle sizes. Composite resins with lower filler size and content are more translucent.

It was observed that, within the shade groups of the composite resins used in this study, total and diffuse transmittance decreased significantly from A shades towards C and D shades in several groups. The highest variations in translucency were observed within the body shades (A to D) of Filtek Supreme and also within the enamel shades of Esthet-X resin. This finding is consistent with a recent study by Ryan et al. who reported that the translucency of Filtek Supreme composite resin significantly affected the color where the boundaries between the enamel and body shade categories were not distinct, and there were some overlaps [24]. These findings emphasize the importance of translucency of composite resins in shade selection. In other words, while selecting a shade for a tooth-colored composite restoration, it is important to realize that the translucency of the resin may decrease significantly towards C and D shades and this can cause dissimilarity between the restoration and natural teeth despite the color match.

The data on the translucency of two multishaded dental composites used in this study provide the clinician with useful information on these highly esthetic restorative materials. However, care must be taken in direct extrapolation of this information to all the other shades produced by the same manufacturer.

Since Filtek Supreme and Esthet.X composite resins have been developed for clinical usage by multi-layering technique, the resulting restorations in this technique is determined by several factors including translucency, color, and thickness of each layer [1]. Therefore accurate knowledge regarding the translucency and color of materials is fundamental to the layering technique. Thus it is important for the clinician to be aware of the optical properties of restorative dental materials such as translucency and color of each product for successful aesthetic restorations.

One of the limitations of this study was that it did not take into account the combined effect of translucency, shade, and the thickness of the layers used. Therefore, in order to optimize the optical properties of multilayered restorative composite restorations, further studies are required to investigate the combined effect of translucency, shade, and thickness of the layers of the restoration.

## 5. Conclusions

From the results of this study it can be concluded that the translucency within each shade category can be severely affected by the color as observed within the body shade group of Filtek Supreme and the enamel shades of Esthet-X composites. However, the variation in translucency within each shade group does not cross over between different shade categories. Provision of information on the translucency of different shades of composite resins can be very useful for the clinicians in selecting the composites with correct shade and translucency.
